# Supplementary material for: Influence of Heating during Cooking on Trans Fatty Acid Content of Edible Oils: A Systematic Review and Meta-Analysis
Source: Nutrients. 2022 Apr 2;14(7):1489. doi: 10.3390/nu14071489 (PMC9002916; doi:10.3390/nu14071489)
Supplement: Supplementary file 1 [file nutrients-14-01489-s001.zip › nutrients-1655757-supplementary.pdf]

Supplementary Table S1: Characteristics of included studies.

Study	Oil	Heating method	Temperature (ºC)	Heating time (min)	Heating cycle	Substrate analysed	Method of analysis	Fatty acid	Baseline TFA level (% of total fatty acids unless stated otherwise)
Grandgirard 1984 [1]	Rapeseed	Heating	200 – 240	600 – 2,400	1	Oil	GC-FID	C18:3t	0.20%
Sebedio 1996 [2]	Peanut	Deep frying	180 – 240	5	1 – 30	Oil, potato	GC-MS	C18:2t	1.60%
	Soybean	Deep frying	180 – 240	5	1 – 30	Oil, potato	GC-MS	C18:2t	0.90%
								C18:3t	9.20%
Tyagi 1996 [3]	Hydrogenated veg. fat	Deep frying	170 – 190	4,200	1	Oil	GC-FID	Total TFA	26.70%
	Soybean	Deep frying	170 – 190	4,200	1	Oil	GC-FID	Total TFA	0.00%
Moreno 1999 [4]	Blend	Heating	80 – 300	20 – 40	1	Oil	FTIR spectroscopy	C18:1t	0.47%
	Corn	Heating	80 – 300	20 – 40	1	Oil	FTIR spectroscopy	C18:1t	0.35%
	Lard	Heating	80 – 300	20 – 40	1	Oil	FTIR spectroscopy	C18:1t	0.79%
	Olive	Heating	80 – 300	20 – 40	1	Oil	FTIR spectroscopy	C18:1t	0.56%
	Sunflower	Heating	80 – 300	20 – 40	1	Oil	FTIR spectroscopy	C18:1t	0.23%
Romero 2000 [5]	Olive	Deep frying	180	8	8 – 20	Oil, potato	GC	C18:1t	0.00%
								C18:2t	0.00%
	Sunflower	Deep frying	180	8	8 – 20	Oil, potato	GC	C18:1t	0.00%
								C18:2t	0.27%
Sanibal & Mancini 2004 [6]	Hydrogenated veg. fat	Deep frying	180	600 – 3,000	20 – 100	Oil	GC-FID	C18:2t	8.56%
								C18:3t	0.14%
	Soybean	Deep frying	180	600 – 3,000	20 – 100	Oil	GC-FID	C18:2t	0.72%
								C18:3t	1.38%
Daniel 2005 [7]	Cottonseed	Deep frying	177	480 – 2,400	1 – 5	Oil, potato	GC	Total TFA	0.10%
	Hydrogenated veg. fat	Deep frying	177	480 – 2,400	1 – 5	Oil	GC-FID	Total TFA	

Liu 2007 [8]	Hydrogenated veg. fat	Heating	160 – 200	240 – 1,440	1	Oil	GC-FID	C16:1t	0.00%
								C18:1t	19.47%
								C18:2t	0.95%
								C18:3t	0.00%
								Total TFA	20.42%
	Soybean	Heating	160 – 200	240 – 1,440	1	Oil	GC-FID	C16:1t	0.00%
								C18:1t	0.00%
								C18:2t	0.00%
								C18:3t	0.00%
								Total TFA	0.00%
Liu 2008 [9]	Hydrogenated veg. fat	Heating	160 – 200	15 – 75	1	Oil, chicken	GC-MS	C16:1t	
								C18:1t	
								C18:2t	
								C18:3t	
								Total TFA	
		Deep frying	160 – 200	15 – 75	1	Oil, chicken	GC-MS	C16:1t	0.00 mg/g chicken
								C18:1t	194.7 mg/g chicken
								C18:2t	9.5 mg/g chicken
								C18:3t	0.00 mg/g chicken
								Total TFA	204.2 mg/g chicken
	Soybean	Deep frying	160 – 200	15 – 75	1	Oil, chicken	GC-MS	C16:1t	0.00 mg/g chicken
								C18:1t	0.00 mg/g chicken
								C18:2t	0.00 mg/g chicken
								C18:3t	0.00 mg/g chicken
								Total TFA	0.00 mg/g chicken

Aladedunya and Przybylski 2009 [10]	Canola	Deep frying	185 – 215	420 – 2,940	8 – 56	Oil	GC	C18:1t	0.10%
								C18:2t	0.70%
								C18:3t	1.55%
								Total TFA	2.45%
Bansal 2009 [11]	Blend	Deep frying	182.5	-	5 – 40	Oil	GC	C18:1t	0.00%
								Total TFA	0.55%
		Heating	182.5	30	5 – 40	Oil	GC	C18:1t	0.00%
								Total TFA	0.54%
	Palm	Deep frying	182.5	-	5 – 40	Oil	GC	C18:1t	0.00%
								Total TFA	0.58%
		Heating	182.5	30	5 – 40	Oil	GC	C18:1t	0.00%
								Total TFA	0.63%
	Sunflower	Deep frying	182.5	-	5 – 40	Oil	GC	C18:1t	0.00%
								Total TFA	0.88%
		Heating	182.5	30	5 – 40	Oil	GC	C18:1t	0.00%
								Total TFA	0.84%
Tena 2009 [12]	Olive	Heating	190	5,640	12	Oil	GC	C16:1t	0.12%
				120 – 5,640	1 – 12	Oil	GC	C18:1t	0.01%
				5,640	12	Oil	GC	C18:2t	0.11%
				5,640	12	Oil	GC	Total TFA	0.13%
Casal 2010 [13]	Olive	Deep frying	170	180 – 1,620	1 – 2	Oil	GC-FID	Total TFA	0.03%
	Sunflower	Deep frying	170	180 – 1,620	1 – 2	Oil	GC-FID	Total TFA	0.27%
Rani 2010 [14]	Hydrogenated veg. fat	Heating	180	480 – 1,440	1	Oil	GC	C18:1t	
	Sunflower	Heating	180	480 – 1,440	1	Oil	GC	C18:1t	0.00%
								C18:2t	0.00%

Tsuzuki 2010 [15]	Blend	Heating	180	120 – 240	1	Oil	GC	C18:1t	0.02%
								C18:2t	0.37%
								C18:3t	1.20%
	Canola	Deep frying	160 – 200	5 – 7	2 – 10	Oil	GC	C18:1t	0.04%
								C18:2t	0.14%
								C18:3t	0.88%
		Heating	160 – 200	100	1	Oil	GC	C18:1t	0.04%
								C18:2t	0.14%
								C18:3t	0.90%
	Corn	Heating	180	120 – 240	1	Oil	GC	C18:1t	0.05%
								C18:2t	1.20%
								C18:3t	0.35%
	Rice bran	Heating	180	120 – 240	1	Oil	GC	C18:1t	0.10%
								C18:2t	0.68%
								C18:3t	0.35%
	Sesame	Heating	180	120 – 240	1	Oil	GC	C18:1t	0.15%
								C18:2t	0.30%
								C18:3t	0.01%
	Safflower	Heating	180	120 – 240	1	Oil	GC	C18:1t	0.05%
								C18:2t	0.19%
								C18:3t	0.05%
	Sunflower	Heating	180	120 – 240	1	Oil	GC	C18:1t	
								C18:2t	
								C18:3t	
Enriquez-Fernández 2011 [16]	Blend	Deep frying	180	2.5	200	Oil	GC-FID	C18:1t	0.06%

								C18:2t	0.06%
	Palm	Deep frying	180	2.5	200	Oil	GC-FID	C18:1t	0.36%
								C18:2t	0.36%
Filip 2011 [17]	Sunflower	Heating	185	960 – 4,800	1 – 5	Oil	GC-FID	C18:1t	0.67%
								C18:2t	0.15%
								Total TFA	0.91%
Kala 2012 [18]	Hydrogenated veg fat	Heating	180	360 – 720	1	Oil	GC	C18:1t	17.00%
								C18:2t	0.70%
								C18:1t	
								C18:2t	
								C18:1t	
								C18:2t	
								C18:1t	
								C18:2t	
	Palm	Heating	180	360 – 720	1	Oil	GC	C18:1t	0.00%
	Peanut	Heating	180	360 – 720	1	Oil	GC	C18:1t	0.00%
Li 2012 [19]	Soybean	Heating	160 – 240	720	1	Oil	GC	C18:1t	0.02%
								Total TFA	0.09%
Yang 2012 [20]	Corn	Heating	140 – 260	15 – 720	1	Oil	GC-FID	C16:1t	0.00%
								C18:1t	0.06%

								C18:2t	0.90%
								Total TFA	0.96%
Li 2013 [21]	Soybean	Heating	180 – 220	720	1	Oil	GC	C18:2t	0.04%
								Total TFA	0.04%
Chen 2014 [22]	Palm	Deep frying	150 – 250	120 – 480	12 – 48	Oil, chicken	GC-FID	C18:1t	
								C18:2t	0.17%
								C18:3t	0.00%
Dhibi 2014 [23]	Aleppo pine seed	Heating	180	120 – 600	1	Oil	GC	C16:1t	0.04%
								C18:1t	0.00%
								C18:2t	0.95%
								C18:3t	0.00%
								C20:1t	0.06%
Mishra and Sharma 2014 [24]	Blend	Deep frying	210	0.13	1 – 6	Oil	GC	Total TFA	1.15%
	Rice brain	Deep frying	210	0.13	1 – 6	Oil	GC	Total TFA	1.27%
Yang 2014 [25]	Corn	Deep frying	170	4	3 – 12	Oil, chicken	GC	C18:1t	0.06%
								C18:2t	0.89%
								C18:3t	0.17%
								Total TFA	1.15%
Zribi 2014 [26]	Corn	Pan frying	180	6	1	Oil	GC	C18:1t	0.02%
		Deep frying	160 – 190	8.5 – 11.5	10	Oil	GC	C18:1t	0.02%
	Olive	Pan frying	180	6	1	Oil	GC	C18:1t	0.03%
		Deep frying	160 – 190	8.5 – 11.5	10	Oil	GC	C18:1t	0.03%
	Soybean	Pan frying	180	6	1	Oil	GC	C18:1t	0.03%
		Deep frying	160 – 190	8.5 – 11.5	10	Oil	GC	C18:1t	0.03%
	Sunflower	Pan frying	180	6	1	Oil	GC	C18:1t	0.02%

		Deep frying	160 – 190	8.5 – 11.5	10	Oil	GC	C18:1t	0.02%
Segura-Campos 2015 [27]	Blend	Deep frying	185	8	1	Oil, fish	GC-MS	C18:1t	2.88%
	Corn	Deep frying	185	8	1	Oil, fish	GC-MS	C18:1t	1.26%
	Safflower	Deep frying	185	8	1	Oil, fish	GC-MS	C18:1t	0.99%
	Sunflower	Deep frying	185	8	1	Oil, fish	GC-MS	C18:1t	
Song 2015 [28]	Corn	Baking	180	2	1	Oil	GC	C18:1t	0.00%
								C18:2t	0.25%
		Stir frying	170	2	1	Oil	GC	C18:1t	0.00%
								C18:2t	0.25%
		Pan frying	170	2	1	Oil	GC	C18:1t	0.00%
								C18:2t	0.25%
		Deep frying	180	2	1	Oil	GC	C18:1t	0.00%
								C18:2t	0.25%
Bhardwaj 2016 [29]	Ghee	Heating	180, 220	30	1, 2	Oil	GC	Total TFA	0.68%
		Deep frying	180, 220	30	1, 2	Oil	GC	Total TFA	0.68%
	Groundnut	Heating	180, 220	30	1, 2	Oil	GC	Total TFA	0.00%
		Deep frying	180, 220	30	1, 2	Oil	GC	Total TFA	0.00%
	Hydrogenated veg. fat	Heating	180, 220	30	1, 2	Oil	GC	Total TFA	13.90%
		Deep frying	180, 220	30	1, 2	Oil	GC	Total TFA	13.90%
	Olive	Heating	180, 220	30	1, 2	Oil	GC	Total TFA	0.00%
		Deep frying	180, 220	30	1, 2	Oil	GC	Total TFA	0.00%
	Rapeseed	Heating	180, 220	30	1, 2	Oil	GC	Total TFA	1.60%
		Deep frying	180, 220	30	1, 2	Oil	GC	Total TFA	1.60%
	Soybean	Heating	180, 220	30	1, 2	Oil	GC	Total TFA	0.00%
		Deep frying	180, 220	30	1, 2	Oil	GC	Total TFA	0.00%

Guo 2016 [30]	Corn	Heating	230	120 – 2,880	-	Oil	GC	-	
Cui 2017 [31]	Corn	Deep frying	175	10 – 720	1 – 48	Oil	GC-MS	C18:1t	
								C18:2t	
								C18:3t	
								Total TFA	0.66%
		Pan frying	160	-	1	Oil	GC-MS	C18:1t	0.03%
								C18:2t	0.60%
								C18:3t	0.37%
								Total TFA	1.00%
		Stir frying	175	5	1	Oil	GC-MS	C18:1t	0.03%
								C18:2t	0.60%
								C18:3t	0.37%
								Total TFA	1.00%
	Linseed	Deep frying	175	10 – 720	1 – 48	Oil	GC-MS	C18:1t	
								C18:2t	
								C18:3t	
								Total TFA	
		Pan frying	160	-	1	Oil	GC-MS	C18:1t	0.01%
								C18:2t	0.07%
								C18:3t	0.04%
								Total TFA	0.12%
		Stir frying	175	5	1	Oil	GC-MS	C18:1t	0.01%
								C18:2t	0.07%
								C18:3t	0.04%
								Total TFA	0.12%
	Olive	Deep frying	175	10 – 720	1 – 48	Oil	GC-MS	C18:1t	

								C18:2t	
								C18:3t	
								Total TFA	
		Pan frying	160	-	1	Oil	GC-MS	C18:1t	0.00%
								C18:2t	0.00%
								C18:3t	0.00%
								Total TFA	0.00%
		Stir frying	175	5	1	Oil	GC-MS	C18:1t	0.00%
								C18:2t	0.00%
								C18:3t	0.00%
								Total TFA	0.00%
	Peanut	Deep frying	175	10 – 720	1 – 48	Oil	GC-MS	C18:1t	
								C18:2t	
								C18:3t	
								Total TFA	0.45%
		Pan frying	160	-	1	Oil	GC-MS	C18:1t	0.00%
								C18:2t	0.08%
								C18:3t	0.10%
								Total TFA	0.17%
		Stir frying	175	5	1	Oil	GC-MS	C18:1t	0.00%
								C18:2t	0.08%
								C18:3t	0.10%
								Total TFA	0.17%
	Peony seed	Deep frying	175	10 – 720	1 – 48	Oil	GC-MS	C18:1t	
								C18:2t	
								C18:3t	

							Total TFA	
	Pan frying	160	-	1	Oil	GC-MS	C18:1t	0.00%
							C18:2t	0.07%
							C18:3t	0.24%
							Total TFA	0.31%
	Stir frying	175	5	1	Oil	GC-MS	C18:1t	0.00%
							C18:2t	0.07%
							C18:3t	0.24%
							Total TFA	0.31%
Rapeseed	Deep frying	175	10 – 720	1 – 48	Oil	GC-MS	C18:1t	
							C18:2t	
							C18:3t	
							Total TFA	0.61%
	Pan frying	160	-	1	Oil	GC-MS	C18:1t	0.02%
							C18:2t	0.26%
							C18:3t	1.19%
							Total TFA	1.47%
	Stir frying	175	5	1	Oil	GC-MS	C18:1t	0.02%
							C18:2t	0.26%
							C18:3t	1.19%
							Total TFA	1.47%
Rice bran	Deep frying	175	10 – 720	1 – 48	Oil	GC-MS	C18:1t	
							C18:2t	
							C18:3t	
							Total TFA	0.72%
	Pan frying	160	-	1	Oil	GC-MS	C18:1t	0.00%

								C18:2t	1.60%
								C18:3t	0.53%
								Total TFA	2.13%
		Stir frying	175	5	1	Oil	GC-MS	C18:1t	0.00%
								C18:2t	1.60%
								C18:3t	0.53%
								Total TFA	2.13%
	Sesame	Deep frying	175	10 – 720	1 – 48	Oil	GC-MS	C18:1t	
								C18:2t	
								C18:3t	
								Total TFA	
		Pan frying	160	-	1	Oil	GC-MS	C18:1t	0.08%
								C18:2t	0.24%
								C18:3t	0.07%
								Total TFA	0.39%
		Stir frying	175	5	1	Oil	GC-MS	C18:1t	0.08%
								C18:2t	0.24%
								C18:3t	0.07%
								Total TFA	0.39%
	Soybean	Deep frying	175	10 – 720	1 – 48	Oil	GC-MS	C18:1t	
								C18:2t	
								C18:3t	
								Total TFA	0.73%
		Pan frying	160	-	1	Oil	GC-MS	C18:1t	0.02%
								C18:2t	0.32%

								C18:3t	0.57%
								Total TFA	0.91%
		Stir frying	175	5	1	Oil	GC-MS	C18:1t	0.02%
								C18:2t	0.32%
								C18:3t	0.57%
								Total TFA	0.91%
	Sunflower	Deep frying	175	10 – 720	1 – 48	Oil	GC-MS	C18:1t	
								C18:2t	
								C18:3t	
								Total TFA	0.40%
		Pan frying	160	-	1	Oil	GC-MS	C18:1t	0.00%
								C18:2t	0.00%
								C18:3t	0.00%
								Total TFA	0.00%
		Stir frying	175	5	1	Oil	GC-MS	C18:1t	0.00%
								C18:2t	0.00%
								C18:3t	0.00%
								Total TFA	0.00%
Li 2017 [32]	Coconut	Deep frying	180	600, 1200, 1800, 2400, 3000	40, 80, 120, 160, 200	Oil	GC	C18:1t	
	Palm	Deep frying	180	600, 1200, 1800, 2400, 3000	40, 80, 120, 160, 200	Oil	GC	C18:1t	
								C18:2t	0.14%
Matar 2019 [33]	Corn	Heating	180, 200, 210, 220	4	1	Oil	GC-MS	C18:1t	0.04%
	Soybean	Heating	180, 200, 210, 220	4	1	Oil	GC-MS	C18:1t	0.06%
	Sunflower	Heating	180, 200, 210, 220	4	1	Oil	GC-MS	C18:1t	0.00%

Supplementary Table S2: Trans fatty acid concentration of two most commonly studied cooking oils (corn and soybean) and hydrogenated vegetable fat at predetermined temperature intervals.

		Trans fatty acid concentration (% of total fatty acids)							
		Unheated		<200°C		200-240°C		>240°C	
Fatty acid	Cooking fat	*n* studies (*n* samples)	Median (IQR)	*n* studies (*n* samples)	Median (IQR)	*n* studies (*n* samples)	Median (IQR)	*n* studies (*n* samples)	Median (IQR)
16:1t	Corn oil	0 (0)	-	1 (24)	0.02 (0.01; 0.03)	1 (16)	0.02 (0.01; 0.03)	1 (16)	0.02 (0.00; 0.02)
	Soybean oil	0 (0)	-	1 (14)	0.02 (0.01; 0.03)	1 (7)	0.03 (0.01; 0.03)	0 (0)	-
	Hydrogenated vegetable fat	0 (0)	-	1 (14)	0.02 (0.02; 0.03)	1 (7)	0.02 (0.02; 0.03)	0 (0)	-
18:1t	Corn oil	2 (3)	0.35 (0.04; 0.35)	5 (34)	0.07 (0.06; 0.08)	3 (21)	0.07 (0.06; 0.15)	2 (20)	0.13 (0.06; 0.70)
	Soybean oil	2 (2)	0.04 (0.02; 0.06)	3 (45)	0.00 (0.00; 0.01)	3 (26)	0.01 (0.00; 0.01)	1 (1)	0.05 (-)
	Hydrogenated vegetable fat	0 (0)	-	3 (74)	5.88 (4.20; 7.13)	1 (21)	5.27 (4.47; 6.15)	0 (0)	-
18:2t	Corn oil	0 (0)	-	3 (77)	0.44 (0.15; 0.49)	1 (48)	0.46 (0.09; 0.56)	1 (48)	0.48 (0.21; 0.98)
	Soybean oil	0 (0)	-	2 (20)	0.01 (0.00; 0.01)	2 (13)	0.01 (0.00; 0.05)	0 (0)	-
	Hydrogenated vegetable fat	0 (0)	-	2 (26)	0.77 (0.35; 0.88)	1 (7)	0.85 (0.76; 0.87)	0 (0)	-
18:3t	Corn oil	0 (0)	-	2 (5)	0.34 (0.17; 0.35)	0 (0)	-	0 (0)	-
	Soybean oil	1 (3)	0.01 (0.00; 0.01)	1 (14)	0.01 (0.00; 0.01)	2 (13)	0.01 (0.00; 0.25)	1 (6)	0.80 (0.65; 1.42)
	Hydrogenated vegetable fat	0 (0)	-	1 (14)	0.00 (0.00; 0.01)	1 (7)	0.00 (0.00; 0.01)	0 (0)	-
Total TFA	Corn oil	0 (0)	-	2 (26)	0.98 (0.96; 1.05)	1 (16)	1.07 (0.96; 1.56)	1 (16)	1.47 (0.96; 3.65)
	Soybean oil	1 (1)	0.09 (-)	4 (21)	0.03 (0.01; 0.06)	4 (14)	0.03 (0.02; 1.14)	1 (1)	7.76 (-)
	Hydrogenated vegetable fat	0 (0)	-	2 (17)	17.9 (16.3; 18.6)	2 (10)	16.8 (14.7; 18.0)	0 (0)	-

Supplementary Table S3: Quality assessment of included studies.

Study	Reporting of analytic variability<br>0 = No<br>1 = Yes	>1 replicates<br>0 = No<br>1 = Yes	Reporting of measures of uncertainty<br>0 = No<br>1 = Yes	Quality score
Grandgirard 1984 [1]	0	0	0	0
Sebedio 1996 [2]	0	0	0	0
Tyagi 1996 [3]	0	0	0	0
Moreno 1999 [4]	0	0	0	0
Romero 2000 [5]	0	1	1	2
Sanibal & Mancini 2004 [6]	0	1	1	2
Daniel 2005 [7]	0	1	0	1
Liu 2007 [8]	1	1	1	3
Liu 2008 [9]	0	1	1	2
Aladedunya and Przybylski 2009 [10]	0	1	1	2
Bansal 2009 [11]	0	1	1	2
Tena 2009 [12]	0	1	0	1
Casal 2010 [13]	0	1	1	2
Rani 2010 [14]	0	1	0	1
Tsuzuki 2010 [15]	0	1	1	2
Enriquez-Fernández 2011 [16]	0	0	1	1
Filip 2011 [17]	0	1	1	2
Kala 2012 [18]	0	1	0	1
Li 2012 [19]	0	1	0	1
Yang 2012 [20]	1	1	1	3
Li 2013 [21]	0	1	0	1

Chen 2014 [22]	1	1	1	3
Dhibi 2014 [23]	0	1	1	2
Mishra and Sharma 2014 [24]	0	1	1	2
Yang 2014 [25]	0	1	1	2
Zribi 2014 [26]	0	1	1	2
Segura-Campos 2015 [27]	0	0	1	1
Song 2015 [28]	0	1	1	2
Bhardwaj 2016 [29]	0	1	1	2
Guo 2016 [30]	0	0	0	0
Cui 2017 [31]	0	1	0	1
Li 2017 [32]	0	1	1	2
Matar 2019 [33]	0	0	0	0

Supplementary Table S4: Summary measures of cooking temperature, cooking time, and cooking cycle according to method of cooking.

Method	Median temperature (ºC) [IQR]	Mean temperature (ºC) (± SD)	Median cooking time (min) [IQR]	Mean cooking time (min) (± SD)	Median cooking cycle [IQR]	Mean cooking cycle (± SD)
Baking	180	180	1 [0 – 2]	1 ± 1.2	1	1
Deep frying	180 [175 – 200]	182 ± 23	30 [5 – 75]	335 ± 747	1 [1 – 12]	14 ± 29
Heating	180 [180 – 200]	187 ± 39	240 [30 – 720]	541 ± 888	1	2.2 ± 5.2
Pan frying	160	164 ± 7.5	2 [0 – 6]	2.8 ± 2.9	1 [0 – 1]	0.5 ± 0.5
Stir frying	175	175 ± 1.1	5	4.8 ± 0.9	1 [0 – 1]	0.5 ± 0.5
All	180 [175 – 200]	183 ± 30	45 [6 – 480]	414 ± 810	1 [1 – 2]	7.9 ± 21

Supplementary Table S5: Summary measures of cooking temperature, cooking time, and cooking cycle according to type of trans fatty acid.

Fatty acid	Median temperature (ºC) [IQR]	Mean temperature (ºC) (± SD)	Median cooking time (min) [IQR]	Mean cooking time (min) (± SD)	Median cooking cycle [IQR]	Mean cooking cycle (± SD)	Median [IQR] baseline level (% of total TFA)
C16:1t	180 [160 – 200]	186 ± 28	60 [15 – 480]	292 ± 556	1	1.1 ± 0.8	0
C18:1t	180 [175 – 190]	180 ± 33	45 [8 – 480]	462 ± 914	1	7.3 ± 24	0.22 [0.04 – 1.15]
C18:2t	180 [175 – 200]	187 ± 32	60 [5 – 480]	414 ± 787	1 [1 – 6]	11 ± 27	0.32 [0.07 – 0.67]
C18:3t	180 [160 – 200]	182 ± 35	60 [5 – 480]	395 ± 689	1 [1 – 6]	8.0 ± 16	0.10 [0 – 0.87]
C20:1t	180	180	300 [120 – 480]	300 ± 224	1	1	0.09 [0.07 – 0.12]
Total TFA	180 [175 – 195]	183 ± 26	30 [10 – 360]	347 ± 758	1 [1 – 2]	5.8 ± 11.3	0.97 [0.41 – 2.01]
All	180 [175 – 200]	183 ± 30	45 [6 – 480]	414 ± 810	1 [1 – 2]	7.9 ± 21	0.34 [0.04 – 1.00]

Supplementary Table S6: Change in TFA content of cooking oils (% of total fatty acids) as a function of heating temperature. Data were fitted using a mixed multilevel linear regression model with random intercepts for studies and splines at 200 and 240ºC. Heating temperature, heating time, and type of oil were specified as fixed effect. Coefficients (ß) represent change in TFA per 10ºC increase in temperature. Model 1: crude, unadjusted. Model 2: adjusted for heating time and oil type.

				**Model 1**		**Model 2**	
**Fatty acid**	**Studies (*n*)**	**Observations (*n*)**	**Temperature (ºC)**	**ß (95% CI)**	***p***	**ß (95% CI)**	***P***
C16:1t	3	104	≤ 200	0.00 (-0.00, 0.00)	0.992	0.00 (-0.00, 0.00)	1.000
			200-240	-0.00 (-0.00, 0.00)	0.984	-0.00 (-0.00, 0.00)	1.000
			>240	0.00 (-0.00, 0.00)	0.998	0.00 (-0.00, 0.00)	1.000
C18:1t	21	632	≤ 200	0.02 (-0.01, 0.05)	0.153	0.02 (-0.01, 0.04)	0.283
			200-240	-0.04 (-0.16, 0.08)	0.536	-0.03 (-0.15, 0.01)	0.567
			>240	0.71 (0.58, 0.84)	<0.001	0.72 (0.59, 0.84)	<0.001
C18:2t	17	498	≤ 200	0.01 (-0.01, 0.03)	0.187	0.01 (-0.01, 0.03)	0.224
			200-240	0.05 (0.01, 0.09)	0.005	0.05 (0.02, 0.08)	0.004
			>240	0.14 (0.06, 0.23)	0.001	0.15 (0.01, 0.23)	0.001
C18:3t	9	220	≤ 200	0.02 (0.01, 0.02)	<0.001	0.02 (0.01, 0.02)	<0.001
			200-240	0.18 (0.14, 0.21)	<0.001	0.18 (0.14, 0.21)	<0.001
			>240	-0.74 (-0.94, -0.54)	<0.001	-0.74 (-0.93, -54)	<0.001
TFA	16	442	≤ 200	0.06 (-0.00, 0.13)	0.058	0.04 (-0.02, 0.11)	0.209
			200-240	0.35 (0.17, 0.53)	<0.001	0.38 (0.20, 0.55)	<0.001
			>240	0.25 (-0.28, 0.77)	0.354	0.24 (-0.28, 0.75)	0.370

Supplementary Table S7: Change in TFA as a function of the interaction between heating temperature and heating time (per 1ºC increase). Data were fitted using mixed multilevel linear regression adjusted for heating time and oil type with random intercepts for studies and splines at 200 and 240ºC.

Fatty acid	Studies (*n*)	Observations *(n)*	Heating temperature (ºC)	Interaction coefficient (95% CI)	*P*
C18:1t	21	583	≤200	-3.73×10^{-6} (-1.25×10^{-5}, 5.04×10^{-6})	0.404
	21	583	200-240	7.65×10^{-6} (-2.19×10^{-5}, 3.72×10^{-5})	0.766
	21	583	>240	-8.19×10^{-5} (-2.05×10^{-4}, 4.14×10^{-5})	0.172
C18:2t	16	184	≤200	4.04×10^{-7} (-4.10×10^{-6}, 4.89×10^{-6})	0.860
	17	478	200-240	2.94×10^{-5} (1.91×10^{-5}, 3.97×10^{-5})	<0.001
	17	478	>240	4.03×10^{-5} (1.13×10^{-5}, 6.92×10^{-5})	<0.001
C18:3t	9	200	≤200	8.38×10^{-6} (5.65×10^{-6}, 1.11×10^{-5})	<0.001
	9	200	200-240	-2.14×10^{-5} (-2.61×10^{-5}, -1.66×10^{-5})	<0.001
	9	200	>240	4.90×10^{-5} (-1.26×10^{-5}, 1.11×10^{-4})	0.248
TFA	16	397	≤200	4.77×10^{-7} (-1.35×10^{-5}, 1.45×10^{-5})	0.947
	16	397	200-240	8.41×10^{-5} (4.18×10^{-5}, 1.26×10^{-4})	<0.001
	16	397	>240	2.05×10^{-4} (1.65×10^{-5}, 3.94×10^{-4})	0.001

Supplementary References

1. Grandgirard, A.; Sebedio, J.L.; Fleury, J. Geometrical isomerization of linolenic acid during heat treatment of vegetable oils. *J. Am. Oil Chem. Soc.* **1984**, *61*, 1563–1568. https://doi.org/10.1007/BF02541633.

2. Sebedio, J.L.; Grandgirard, A.; Prevost, J. Linoleic acid isomers in heat treated sunflower oils. *J. Am. Oil Chem. Soc.* **1988**, *65*, 362–366. doi:doi.org/10.1007/BF02663077.

3. Tyagi, V.K.; Vasishtha, A.K. Changes in the characteristics and composition of oils during deep-fat frying. *J. Am. Oil Chem. Soc.* **1996**, *73*, 499–506. https://doi.org/10.1007/BF02523926.

4. Moya Moreno, M.C.M.; Mendoza Olivares, D.; Amézquita López, F.J.; Gimeno Adelantado, J.V.; Bosch Reig, F. Determination of unsaturation grade and trans isomers generated during thermal oxidation of edible oils and fats by FTIR. *J. Mol. Struct.* **1999**, *482–483*, 551–556. https://doi.org/10.1016/S0022-2860(98)00937-5.

5. Romero, A.; Cuesta, C.; Sánchez-Muniz, F.J. Trans fatty acid production in deep fat frying of frozen foods with different oils and frying modalities. *Nutr. Res.* **2000**, *20*, 599–608. https://doi.org/10.1016/S0271-5317(00)00150-0.

6. Sanibal, E.A.A.; Mancini Filho, J. Perfil de ácidos graxos trans de óleo e gordura hidrogenada de soja no processo de fritura. *Ciêne Tecnol. Aliment.* **2004**, *24*, 27–31.

7. Daniel, D.R.; Thompson, L.D.; Shriver, B.J.; Wu, C.-K.; Hoover, L.C. Nonhydrogenated cottonseed oil can be used as a deep fat frying medium to reduce trans-fatty acid content in french fries. *J. Am. Diet. Assoc.* **2005**, *105*, 1927–1932. https://doi.org/10.1016/j.jada.2005.09.029.

8. Liu WH, Stephen Inbaraj B, Chen BH. Analysis and formation of trans fatty acids in hydrogenated soybean oil during heating. *Food Chem.* **2007**, *104*, 1740–1749. https://doi.org/10.1016/j.foodchem.2006.10.069.

9. Liu, W.H.; Lu, Y.F.; Inbaraj, B.S.; Chen, B.H. Formation of trans fatty acids in chicken legs during frying. *Int. J. Food Sci. Nutr.* **2008**, *59*, 368–382. https://doi.org/10.1080/09637480701580645.

10. Aladedunye, F.; Przybylski, R. Degradation and nutritional quality changes of oil during frying. *J. Am. Oil Chem. Soc.* **2008**, *86*, 149–156. https://doi.org/10.1007/s11746-008-1328-5.

11. Bansal, G.; Zhou, W.; Tan, T.-W.; Neo, F.-L.; Lo, H.-L. Analysis of trans fatty acids in deep frying oils by three different approaches. *Food Chem.* **2009**, *116*, 535–541. https://doi.org/10.1016/j.foodchem.2009.02.083.

12. Tena, N.; Aparicio, R.; Garcia-Gonzalez, D.L. Thermal deterioration of virgin olive oil monitored by ATR-FTIR analysis of trans content. *J. Agric. Food Chem.* **2009**, *57*, 9997–10003. https://doi.org/10.1021/jf9012828.

13. Casal, S.; Malheiro, R.; Sendas, A.; Oliveira, B.P.; Pereira, J.A. Olive oil stability under deep-frying conditions. *Food Chem. Toxicol.* **2010**, *48*, 2972–2979. https://doi.org/10.1016/j.fct.2010.07.036.

14. Rani, A.K.S.; Reddy, S.Y.; Chetana, R. Quality changes in trans and trans free fats/oils and products during frying. *Eur. Food Res. Technol.* **2010**, *230*, 803–811. https://doi.org/10.1007/s00217-010-1225-7.

15. Tsuzuki, W.; Matsuoka, A.; Ushida, K. Formation of trans fatty acids in edible oils during the frying and heating process. *Food Chem.* **2010**, *123*, 976–982. https://doi.org/10.1016/j.foodchem.2010.05.048.

16. Enríquez-Fernández, B.E.; Álvarez de la Cadena y Yañez, L.; Sosa-Morales, M.E. Comparison of the stability of palm olein and a palm olein/canola oil blend during deep-fat frying of chicken nuggets and French fries. *Int. J. Food Sci. Technol.* **2011**, *46*, 1231–1237. https://doi.org/10.1111/j.1365-2621.2011.02627.x.

17. Filip, S.; Hribar, J.; Vidrih, R. Influence of natural antioxidants on the formation of trans-fatty-acid isomers during heat treatment of sunflower oil. *Eur. J. Lipid Sci. Technol.* **2010**, *113*, 224–230. https://doi.org/10.1002/ejlt.200900231.

18. Kala, A.L.A.; Joshi, V.; Gurudutt, K.N. Effect of heating oils and fats in containers of different materials on their trans fatty acid content. *J. Sci. Food Agric.* **2012**, *92*, 2227–2233. https://doi.org/10.1002/jsfa.5638.

19. Li, A.; Ha, Y.; Wang, F.; Li, W.; Li, Q. Determination of thermally induced trans-fatty acids in soybean oil by attenuated total reflectance fourier transform infrared spectroscopy and gas chromatography analysis. *J. Agric. Food Chem.* **2012**, *60*, 10709–10713. https://doi.org/10.1021/jf3033599.

20. Yang, M.; Yang, Y.; Nie, S.; Xie, M.; Chen, F. Analysis and formation of trans fatty acids in corn oil during the heating process. *J. Am. Oil Chem. Soc.* **2011**, *89*, 859–867. https://doi.org/10.1007/s11746-011-1974-x.

21. Li, A.; Yuan, B.; Li, W.; Wang, F.; Ha, Y. Thermally induced isomerization of linoleic acid in soybean oil. *Chem. Phys. Lipids* **2012**, *166*, 55–60. https://doi.org/10.1016/j.chemphyslip.2012.12.003.

22. Chen, Y.; Yang, Y.; Nie, S.; Yang, X.; Wang, Y.; Yang, M.; Li, C.; Xie, M. The analysis of trans fatty acid profiles in deep frying palm oil and chicken fillets with an improved gas chromatography method. *Food Control* **2014**, *44*, 191–197. https://doi.org/10.1016/j.foodcont.2014.04.010.

23. Dhibi, M.; Issaoui, M.; Brahmi, F.; Mechri, B.; Mnari, A.; Cheraif, I.; Skhiri, F.; Gazzah, N.; Hammami, M. Nutritional quality of fresh and heated Aleppo pine (Pinus halepensis Mill.) seed oil: Trans-fatty acid isomers profiles and antioxidant properties. *J. Food Sci. Technol.* **2012**, *51*, 1442–1452. https://doi.org/10.1007/s13197-012-0664-5.

24. Mishra, R.; Sharma, H.K. Effect of frying conditions on the physico-chemical properties of rice bran oil and its blended oil. *J. Food Sci. Technol.* **2011**, *51*, 1076–1084. https://doi.org/10.1007/s13197-011-0602-y.

25. Yang, M.; Yang, Y.; Nie, S.; Xie, M.; Chen, F.; Luo, P.G. Formation of trans fatty acids during the frying of chicken fillet in corn oil. *Int. J. Food Sci. Nutr.* **2014**, *65*, 306–310. https://doi.org/10.3109/09637486.2013.858237.

26. Zribi, A.; Jabeur, H.; Aladedunye, F.; Rebai, A.; Matthaus, B.; Bouaziz, M. Monitoring of quality and stability characteristics and fatty acid compositions of refined olive and seed oils during repeated pan- and deep-frying using GC, FT-NIRS, and chemometrics. *J. Agric. Food Chem.* **2014**, *62*, 10357–10367. https://doi.org/10.1021/jf503146f.

27. Segura-Campos, M.; Gonzalez-Barrios, G.; Acereto-Escoffie, P.; Rosado-Rubio, G.; Chel-Guerrero, L.; Betancur-Ancona, D. Fatty acid profile of mero (*Epinephelus morio*) raw and processed oil captured in the Yucatan Peninsula, Mexico. *Nutr. Hosp.* **2014**, *31*, 928–935. https://doi.org/10.3305/nh.2015.31.2.7735.

28. Song, J.; Park, J.; Jung, J.; Lee, C.; Gim, S.Y.; Ka, H.; Yi, B.; Kim, M.-J.; Kim, C.-I.; Lee, J. Analysis of trans fat in edible oils with cooking process. *Toxicol. Res.* **2015**, *31*, 307–312. https://doi.org/10.5487/TR.2015.31.3.307.

29. Bhardwaj, S.; Passi, S.J.; Misra, A.; Pant, K.K.; Anwar, K.; Pandey, R.M.; Kardam, V. Effect of heating/reheating of fats/oils, as used by Asian Indians, on trans fatty acid formation. *Food Chem.* **2016**, *212*, 663–670. https://doi.org/10.1016/j.foodchem.2016.06.021.

30. Guo, Q.; Wang, F.; He, F.; Ha, Y.-M.; Li, Q.-P.; Jin, J.; Deng, Z.-X. The impact of technical cashew nut shell liquid on thermally-induced trans isomers in edible oils. *J. Food Sci. Technol.* **2015**, *53*, 1487–1495. https://doi.org/10.1007/s13197-015-2147-y.

31. Cui, Y.; Hao, P.; Liu, B.; Meng, X. Effect of traditional Chinese cooking methods on fatty acid profiles of vegetable oils. *Food Chem.* **2017**, *233*, 77–84. https://doi.org/10.1016/j.foodchem.2017.04.084.

32. Li, X.; Li, J.; Wang, Y.; Cao, P.; Liu, Y. Effects of frying oils' fatty acids profile on the formation of polar lipids components and their retention in French fries over deep-frying process. *Food Chem.* **2017**, *237*, 98–105. https://doi.org/10.1016/j.foodchem.2017.05.100.

33. Matar, R.; Salami, M.; Al Assaf, Z. Factors affecting the formation of elaidic acid in syrian edible oils during frying with home conditions. *Res. J. Pharm. Technol.* **2019**, *12*, 5451. https://doi.org/10.5958/0974-360X.2019.00945.4.
